# Targeting EGFR/IGF-IR Functional Crosstalk in 2D and 3D Triple-Negative Breast Cancer Models to Evaluate Tumor Progression

**DOI:** 10.3390/ijms26178665

**Published:** 2025-09-05

**Authors:** Spyros Kremmydas, Chrisavgi Gourdoupi, Zoi Piperigkou, Nikos K. Karamanos

**Affiliations:** Biochemistry, Biochemical Analysis & Matrix Pathobiology Research Group, Laboratory of Biochemistry, Department of Chemistry, University of Patras, 26504 Patras, Greece; up1073639@ac.upatras.gr (S.K.); up1061190@ac.upatras.gr (C.G.); zoipip@upatras.gr (Z.P.)

**Keywords:** breast cancer, 3D spheroids, EGFR, IGF-IR, 2D vs. 3D cell cultures

## Abstract

Breast cancer is the most prevalent solid tumor diagnosed in women worldwide, remaining a leading cause of cancer-related mortality. Among its subtypes, triple-negative breast cancer (TNBC) is characterized by high aggressiveness and heterogeneity, accounting for approximately 90% of breast cancer-related deaths. Receptor tyrosine kinases (RTKs), such as epidermal growth factor receptor (EGFR) and the insulin-like growth factor I receptor (IGF-IR), are critical cell growth and survival regulators, with their dysregulation closely related to therapy resistance in breast cancer. Studies on RTK targeting have shown promise, and recently attention has shifted toward developing more physiologically relevant preclinical models. Unlike traditional two-dimensional (2D) cell cultures, 3D models such as spheroids better mimic the complex nature of the tumor microenvironment (TME), offering a more accurate representation of tumor behavior and progression. This study utilized both 2D and 3D culture models to assess the effects of EGFR and IGF-IR inhibition, individually and in combination, in two TNBC cell lines with distinct metastatic potential. The results demonstrate that both receptors play critical roles in regulating key cellular functions, including migration, expression of epithelial-to-mesenchymal transition (EMT) markers and matrix metalloproteinases (MMPs). The use of 3D spheroid models enabled the evaluation of additional functional properties, such as spheroid growth and dissemination, revealing treatment-dependent responses to combined receptor inhibition. Overall, this dual-model approach underscores the importance of incorporating 3D culture systems in preclinical cancer research and provides new insights into the regulatory roles of EGFR and IGF-IR in TNBC progression.

## 1. Introduction

Breast cancer is the most commonly diagnosed cancer in women worldwide and remains a leading cause of cancer-related deaths [1,2]. This disease can be further classified into five main subtypes based on the expression levels of molecular markers like the estrogen receptors (ERs; ERα and ERβ), the progesterone receptor (PR), the human epidermal receptor 2 (HER2) and the recently added proliferation marker Ki-67 [3,4]. Among these, TNBC is the most aggressive subtype lacking ERα, PR, and HER2, although expressing ERβ. Particularly, it accounts for 15–20% of breast cancer cases and is strongly linked to *BRCA1/2* mutations, showing high recurrence, poor prognosis, and significant molecular and cellular heterogeneity. Additionally, due to the absence of hormonal and HER2 targets, treatment options remain limited and challenging [5,6,7]. Recent research has shown that cancer progression is not only driven by genetic changes in tumor cells but is also significantly influenced by the surrounding TME [8]. A key part of the TME is the extracellular matrix (ECM), a dynamic 3D network that not only provides physical support but also regulates cellular functions through chemical and mechanical stimuli [9,10]. In breast cancer, changes in ECM composition, stiffness, and organization are linked to enhanced cancer cell proliferation, migration, and invasion, and are closely tied to poor prognosis. Specific components like matrix remodeling enzymes contribute to these changes and can promote biological processes like EMT, further leading to increased cancer cell motility. Therefore, understanding how tumor cells interact with the surrounding microenvironment is crucial for developing effective treatments [11,12,13,14]. Among the main molecular agents in these interactions are also RTKs, including EGFR and IGF-IR, which are vital regulators of basic cellular functions like proliferation, survival and differentiation [15]. In cancer, dysregulation of RTKs leads to constant activation and disruption in the equilibrium between cell growth and cell death, promoting cancer progression [16,17]. Moreover, EGFR and IGF-IR are key players in cancer progression, triggering signaling cascades that influence multiple cellular behaviors such as cell survival and proliferation, metabolic reprogramming, resistance to apoptosis, EMT, invasion and metastasis [18,19]. Clinical evidence indicates that EGFR overexpression is linked to poorer prognosis and adverse outcomes in aggressive TNBC patients, while IGF-IR is overexpressed in varying degrees across breast cancer subtypes [20,21]. Due to their important role in cancer progression, RTKs have become prominent targets for molecular therapies, with both monoclonal antibodies (mAbs), designed to block ligand binding or receptor dimerization, and small chemical molecule inhibitors, aimed at competitively occupying the ATP-binding site, being developed. Although these strategies have led to notable clinical advances, their long-term efficacy is often limited by adverse side effects, insufficient specificity, and drug resistance [17,22].

MMPs are key regulators of tissue homeostasis that support processes like wound healing and organ development by remodeling the ECM through the degradation of structural proteins such as collagens, fibronectin, and laminins [23,24]. Dysregulated MMP activity is associated with various pathological conditions, including cancer, where certain MMPs influence tumor progression in a context-dependent manner by shaping TME. Through ECM degradation and modulation of growth factor availability to their receptors, MMPs contribute to key cancer processes such as proliferation, invasion, migration and angiogenesis, with altered expression reported in multiple cancer types including breast, lung, liver, and colorectal [25,26,27].

Much of our understanding of these molecular pathways has been derived from conventional 2D cell cultures, which provide a cost-effective, simple, and easily reproducible model [28]. Such 2D models are also frequently used to evaluate imaging probes and drug responses, as shown in recent theranostic studies [29]. However, 2D models do not accurately emulate the complex architecture and biological context of solid tumors, offering limited insights into how tumor cells behave in a more physiologically relevant 3D environment. In contrast, 3D culture models, particularly spheroids, have emerged as valuable models in cancer research. These systems better replicate tumor characteristics such as oxygen and nutrient gradients, promoting natural cell–cell and cell–matrix interactions [30,31,32]. Importantly, spheroids can produce their own ECM, enabling a more biologically relevant TME. They also exhibit different gene and protein expression levels associated with cancer progression, including EMT markers and receptors (e.g., EGFR). At the same time, the transition from 2D to 3D cultures limits direct cell targeting and creates hypoxic inner layers, altering metabolism and promoting drug resistance. Thus, 3D spheroid cultures serve as a more accurate and functional bridge between in vitro and in vivo platforms, advancing both cancer biology research and drug screening [30,33,34].

This study aimed to elucidate the role of EGFR and IGF-IR in TNBC by examining the effects of their inhibition on cellular functions and gene expression in two distinct TNBC cell lines: the highly aggressive Hs578T (low-ERβ) and the epithelial-like shERβ MDA-MB-231 (ERβ-suppressed). Both conventional 2D cultures and 3D spheroid models were employed to comprehensively assess the effects of the EGFR downstream inhibitor, AG1478, and the IGF-IR downstream inhibitor, AG1024, both individually and in combination. Initially, 3D spheroid models were developed for both cell lines to investigate the inhibitors’ influence on spheroid growth, dissemination, and the migratory capacity of spheroid-derived cells in comparison with their 2D counterparts. Bioinformatic analysis was performed to explore potential interaction networks among EGFR, IGF-IR, EMT markers, and MMPs, while both 2D and 3D culture systems were used to assess gene expression changes following receptor inhibition, providing a multifaceted understanding of the molecular and functional impact of RTKs in TNBC progression.

## 2. Results

### 2.1. Development of 3D TNBC Cell-Derived Spheroids

**Figure 1:**
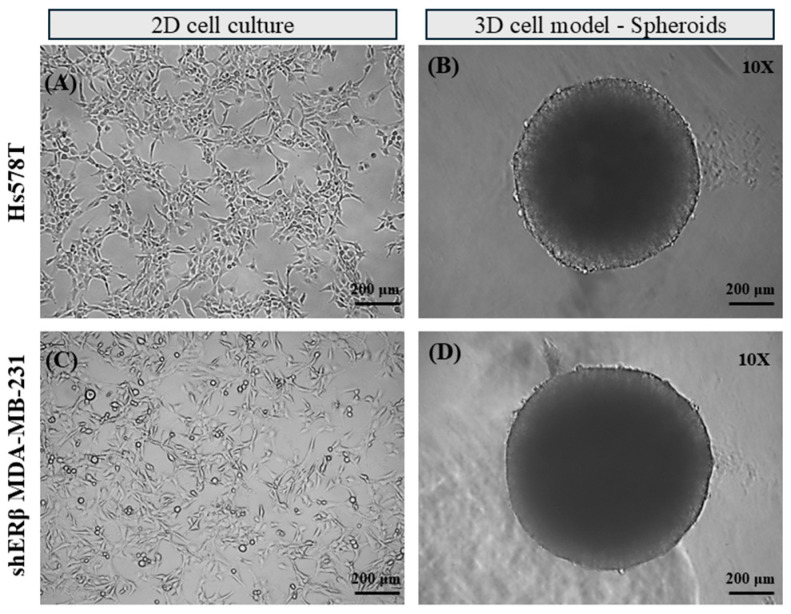
Development and morphological characteristics of breast cancer cell-derived spheroids. Representative images of the highly aggressive Hs578T (**A**) and epithelial-like shERβ MDA-MB-231 (**C**) cells, cultured in 2D monolayers, as well as Hs578T-derived (**B**) and shERβ MDA-MB-231-derived (**D**) spheroids, developed at day 3 (72 h) in ultra-low-adhesion plates. Both cell lines formed well-organized and compacted spheroids, despite their different morphologies in 2D monolayers.

To evaluate tumor cancer cell properties simulating the complex in vivo solid tumor conditions, 3D culture models were developed to investigate the effects of inhibiting EGFR and IGF-IR in TNBC cells. Specifically, the Hs578T and shERβ MDA-MB-231 cell lines were cultivated for 72 h in 96-well ultra-low-adhesive round-bottom plates to facilitate 3D spheroid formation. Figure 1 illustrates representative images of both cell lines in 2D (Figure 1A,C) and 3D conditions (Figure 1B,D), highlighting their morphological similarities and differences. Notably, despite their distinct characteristics in 2D monolayers, with Hs578T demonstrating a mesenchymal and more aggressive phenotype, and shERβ MDA-MB-231 exhibited an epithelial-like phenotype of lower aggressiveness, both cell lines developed similarly structured spheroids with high cellular density and compactness. Notably, shERβ MDA-MB-231 spheroids reached an average diameter of approximately 900 μm, whereas Hs578T spheroids were slightly smaller, averaging around 800 μm.

### 2.2. Effect of EGFR/IGF-IR Inhibition on Spheroid Growth Rate

Assays measuring growth rates in 3D environments allow for the evaluation of treatment impacts on tumor-like formations over time, offering valuable information on cell proliferation, survival, and responses to therapies. By capturing intricate behaviors such as the growth rate of spheroids, these assays are important tools in preclinical cancer research [35,36,37]. To achieve this, the developed spheroids derived from Hs578T and shERβ MDA-MB-231 cells were treated with the inhibitors AG1478, AG1024, and their combination (mixed AGs) to assess changes in their growth rates after 24 h. Furthermore, spheroid-derived cells were analyzed for the effect of the inhibitors on their proliferation, through manual counting, following 24 h of treatment.

**Figure 2:**
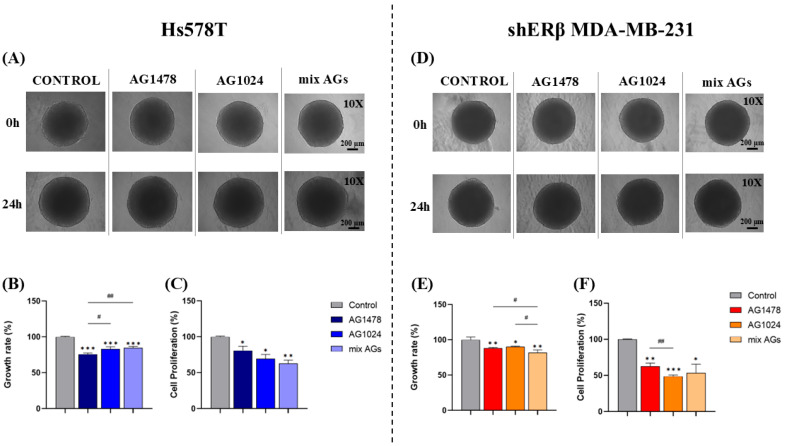
The impact of EGFR and IGF-IR inhibition on spheroid growth rate (**B**,**E**) and spheroid-derived cell proliferation (**C**,**F**). Spheroids derived from Hs578T and shERβ MDA-MB-231 cells were cultured for 72 h and were then treated for 24 h with the inhibitors of EGFR and IGF-IR, AG1478 and AG1024, as well as with their mixture (AG1478 + AG1024), in final concentrations of 2 μΜ and 1 μΜ, respectively. (**A**,**D**) Representative images of Hs578T and shERβ MDA-MB-231 spheroids at time points 0 and 24 h (scale bar 200 μm; magnification 10×). (**B**,**E**) Quantification graphs of Hs578T and shERβ MDA-MB-231 spheroid growth rates after 24 h of treatment in comparison with the control samples. (**C**,**F**) Quantification graphs of Hs578T and shERβ MDA-MB-231 spheroid-derived cell proliferation after 24 h of treatment in comparison with the control samples. Each bar represents mean ± SD value triplicates for each treatment condition. An asterisk (*) indicates statistically significant differences (*p* < 0.05), two asterisks (**) indicate statistically significant differences (*p* < 0.01) and three asterisks (***) indicate statistically significant differences (*p* < 0.001) compared to the untreated spheroids (control). A hash (#) indicates statistically significant differences (*p* < 0.05), two hashes (##) indicate statistically significant differences (*p* < 0.01) between the different treatments.

More precisely, in Hs578T, all treatments led to a similar decrease in growth rate with a reduction of approximately 20% in each sample (Figure 2A,B). In shERβ MDA-MB-231, the inhibition of EGFR and IGF-IR separately led to a decrease in spheroid growth rate (*ca* 10%) with a higher reduction to be obtained using the combination of both inhibitors (*ca* 20%) (Figure 2D,E). Assessing cell proliferation with manual counting of the cells (Figure 2C,F) showed that all treatments led to a significant reduction in cell proliferation in both cell lines. Overall, the observed changes in growth rate support the critical involvement of EGFR and IGF-IR in sustaining tumor cell proliferation under 3D conditions.

### 2.3. Effect of EGFR/IGF-IR Inhibition on Spheroid Dissemination

Metastasis is a complicated, multistep process in which only a small number of circulating tumor cells can adapt and develop secondary tumors. In the early stages of this process, cells experience EMT, altering their phenotypic traits and facilitating their dissemination, which is a crucial characteristic for understanding cancer progression [38]. To further investigate this property in breast cancer cells, spheroids were transferred from U-shaped 96-well plates to flat-bottom plates after the treatment with the inhibitors. Then they were observed for 96 h, and representative photos of their dissemination abilities were captured every 24 h, compared to untreated cells (control).

**Figure 3:**
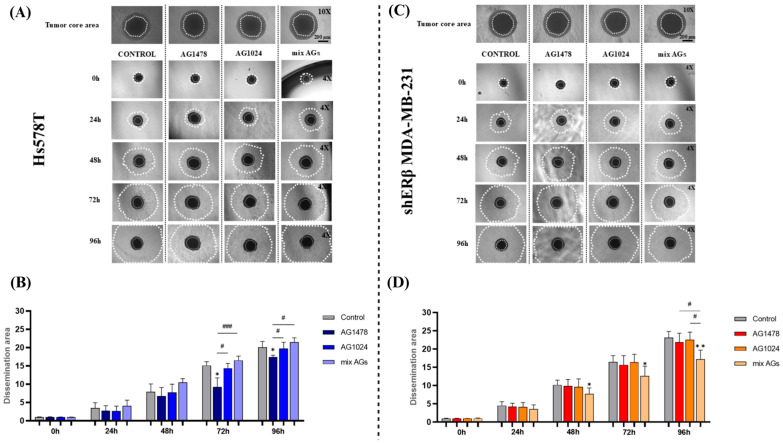
Evaluation of the effect of AG1478, AG1024 and their mixture on the dissemination of Hs578T and shERβ MDA- MB-231 spheroids as models to study the initial steps of tumor metastasis. (**A**,**C**) Representative images of Hs578T- and shERβ MDA-MB-231-derived spheroid tumor core area (scale bar 200 μm; magnification 10×) and spheroid dissemination on polystyrene well plates at the timepoints of 0, 24, 48, 72 and 96 h (magnification 4×). (**B**,**D**) Quantification graphs of the dissemination areas of Hs578T and shERβ MDA- MB-231 spheroids. Each bar represents mean ± SD values in triplicate for each treatment condition. An asterisk (*) indicates statistically significant differences (*p* < 0.05) and two asterisks (**) indicate statistically significant differences (*p* < 0.01), compared to the untreated spheroids (control). A hash (#) indicates statistically significant differences (*p* < 0.05), while three hashes (###) indicate statistically significant differences (*p* < 0.001) between the different treatments in each timepoint.

Interestingly, as it is seen in Figure 3, both cell lines transitioned from their 3D organizational structures to 2D morphologies within the first 24 h. Notably, Hs578T spheroids demonstrated a response to EGFR inhibition, showing a reduction in the dissemination area after 72 and 96 h compared to the untreated spheroids. On the other hand, cells from the shERβ MDA-MB-231 spheroids seem to respond to the combination treatment (mix AGs), with the dissemination area visibly shrinking compared to control cells after 48 h, a pattern that remains consistent after 72 and 96 h as well. The findings emphasize the benefits of utilizing spheroids for assessing treatment outcomes in the initial steps of tumor metastasis. Importantly, both receptors appear as key players in this functional characteristic of highly metastatic cancer cells.

### 2.4. Evaluation of the Effect of EGFR/IGF-IR Inhibition on the Migratory Capacity of Spheroid-Derived Cells vs. 2D Cell Cultures

A defining feature of cancer progression is the enhanced ability of cancer cells to migrate and invade surrounding tissues, ultimately leading to metastasis. After the evaluation of the spreading of spheroid-derived cells, a wound healing assay took place to determine their migration capability after receptor inhibition. Additionally, the same method was conducted in 2D monolayers under the same treatment conditions to evaluate the differences in treatment response in comparison with spheroid-derived cells.

**Figure 4:**
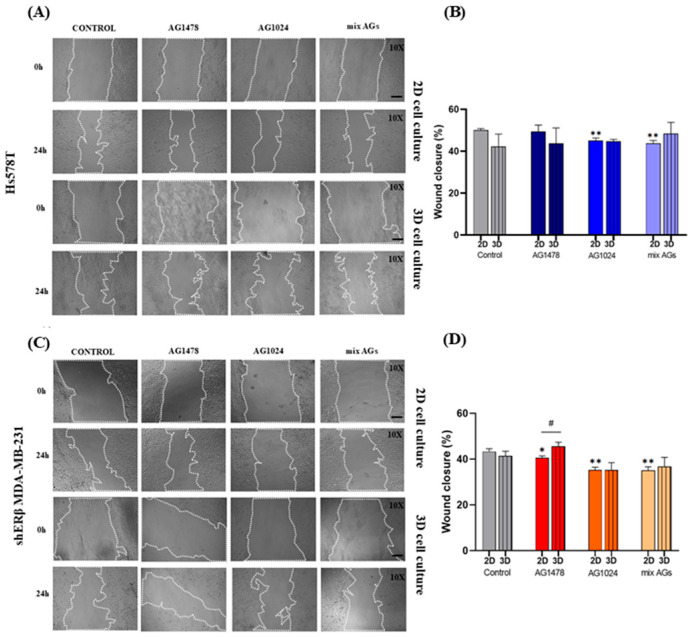
Comparison of 2D and 3D migratory capacities of Hs578T and shERβ MDA-MB-231 cells under EGFR and IGF-IR inhibition. (**A**,**C**) Representative images of the wound healing assays of Hs578T and shERβ MDA-MB-231 in 2D and 3D spheroid-derived cells at 0 h and 24 h time points after treatment with the inhibitors AG1478, AG1024 and their mixture (scale bar 200 μΜ; magnification 10×). (**B**,**D**) Quantification graphs of Hs578T and shERβ MDA-MB-231 spheroid-derived cell (bars with black lines) wound closure after 24 h incubation with the treatments, in comparison with their corresponding treatment in 2D monolayers. Each bar represents mean ± SD values in triplicate for each treatment condition. An asterisk (*) indicates statistically significant differences (*p* < 0.05), and two asterisks (**) indicate statistically significant differences (*p* < 0.01), compared to the untreated cells (control) under 2D or 3D conditions. A hash (#) indicates statistically significant differences (*p* < 0.05) between 2D and 3D samples under the same treatment conditions.

From the results obtained in 2D monolayer cultures (Figure 4), IGF-IR inhibition led to a decrease (*ca* 10%) in the migration of Hs578T cells, an effect also observed under the combined inhibition treatment. Similarly, a notable decrease in cell migration was observed in shERβ MDA-MB-231 cells across all experimental conditions. The most significant reduction (*ca* 20%) occurred following IGF-IR inhibition and the combined treatment. In contrast, under 3D culture conditions, the association between the two receptors and the migratory capacity appeared more complex and varied across treatment types, without showing statistically significant differences compared to the untreated control cells. However, comparative analysis between 2D and 3D cultures under the same treatment conditions, as shown in the quantification graphs with hashed lines (Figure 4), revealed that shERβ MDA-MB-231 spheroid-derived cells exhibited slightly increased wound closure (*ca* 5%) following EGFR inhibition in comparison to their 2D counterparts. The latter might suggest that spheroid-derived cells are less affected by the treatment, likely due to distinct cellular responses in 3D conditions as opposed to 2D monolayers.

### 2.5. Protein–Protein Interactions of EGFR/IGF-IR with EMT Markers and Matrix Remodeling Enzymes

**Figure 5:**
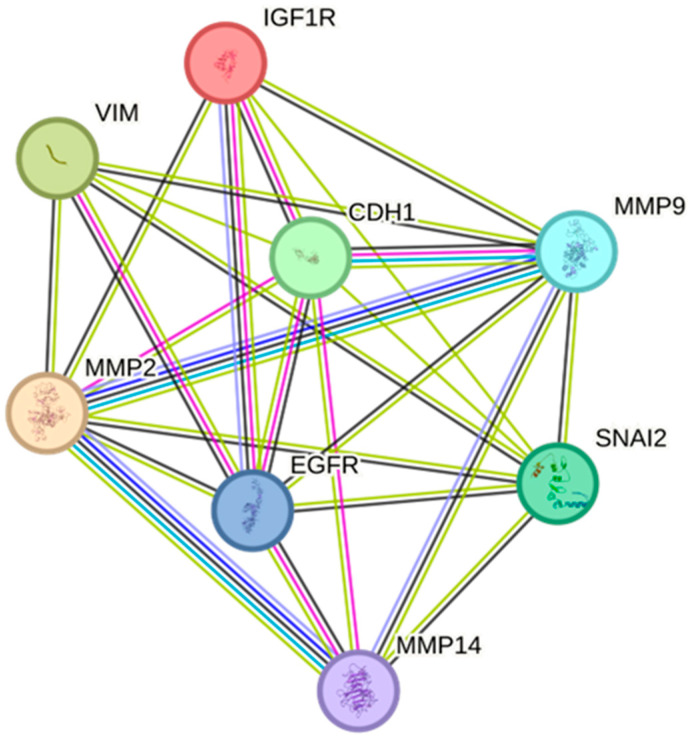
Protein–protein interaction network of EGFR and IGF-IR receptors with MMPs and EMT markers. The interaction network from the STRING database involving EGFR, IGF-IR (IGF1R), MMPs, and major EMT markers (E-cadherin, CDH1; vimentin, VIM, and SNAI2/Slug, SNAI2) was constructed using the Multiple Proteins function. Nodes represent individual proteins, and different line colors indicate known, predicted and other interaction types.

Following the assessment of EGFR and IGF-IR inhibition on the functional characteristics of Hs578T and shERβ MDA-MB-231 cells in both models, a protein–protein interaction network was established using the STRING database to evaluate the physical and functional interactions among EGFR and IGF-IR with MMPs and EMT markers. This network comprised MMP-2, -9, -14, and EMT markers including SNAI2/Slug, vimentin (VIM), and E-cadherin (CDH1). As illustrated in Figure 5, a significant experimentally validated interaction (indicated by purple lines) was noted between EGFR and IGF-IR. Importantly, EGFR also exhibits direct interactions and strong co-expression (shown by black lines) with all the components analyzed, emphasizing its pivotal role in regulating ECM-related pathways in the TME. In addition, IGF-IR demonstrates direct interactions with MMP-2, MMP-9, and CDH1. Collectively, these findings highlight EGFR and IGF-IR as key modulators of ECM dynamics and EMT, underscoring their critical roles in TNBC progression. This prompted us to further investigate the expression of major matrix effectors associated with EGFR and IGF-IR.

### 2.6. EGFR/IGF-IR Inhibition Effects on Gene Expression of EMT Markers and MMPs in 2D and 3D TNBC Cell Cultures

**Figure 6:**
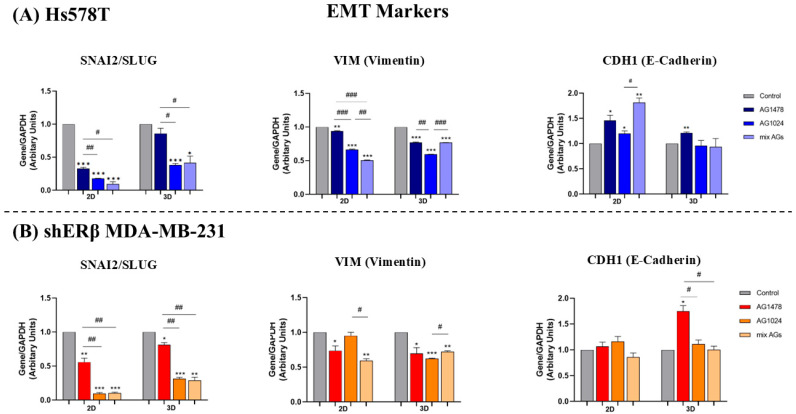
Effect of EGFR and IGF-IR inhibition on gene expression levels of EMT markers in Hs578T and shERβ MDA-MB-231 in 2D and 3D cell cultures. (**A**) Quantification graphs of real-time PCR analysis of *SNAI2*, *VIM* and *CDH1* mRNA levels in 2D and 3D cell cultures after treatment with the inhibitors AG1478, AG1024 and their combination in Hs578T cells. (**B**) Quantification graphs of real-time PCR analysis of *SNAI2*, *VIM* and *CDH1* mRNA levels in 2D and 3D cell cultures after treatment with the inhibitors AG1478, AG1024 and their combination in shERβ MDA-MB- 231 cells. Each bar represents mean ± SD values from triplicate samples. An asterisk (*) indicates statistically significant differences (*p* < 0.05), two asterisks (**) indicate statistically significant differences (*p* < 0.01) and three asterisks (***) indicate statistically significant differences (*p* < 0.001) compared to the control samples. A hash (#) indicates statistically significant differences (*p* < 0.05), two hashes (##) indicate statistically significant differences (*p* < 0.01) and three hashes (###) indicate statistically significant differences between the different samples.

To elucidate the molecular mechanisms associated with the observed functional alterations, we evaluated the expression levels of major EMT markers following EGFR and IGF-IR inhibition in both 2D and 3D culture models. In particular, the expression levels of SNAI2/Slug and vimentin (VIM), which are crucial regulators of mesenchymal transition in breast cancer, were evaluated after treatment with each inhibitor as well as their combination. The epithelial marker E-Cadherin (CDH1) was also included in this analysis [39,40]. In Hs578T cells (Figure 6A), inhibition of either receptor alone or their combination led to decreased *SNAI2* and *VIM* expression, while *CDH1* levels were maintained or increased under both 2D and 3D conditions, consistent with probable crosstalk between the receptors. In the shERβ MDA-MB-231 cells (Figure 6B), a significant reduction in *SNAI2* and *VIM* expression was observed following all treatments. Specifically, under 2D conditions, *SNAI2* and *VIM* exhibited a notable decrease after the combination treatment, aligning with the results of the migratory capacity, suggesting a decrease in the aggressiveness of this cell line. Furthermore, *CDH1* expression remained unchanged, with no statistically significant differences when compared to control samples. Notably, 3D cultures displayed a similar expression pattern to their 2D counterparts, with EGFR inhibition also leading to an increase in *CDH1* expression, further supporting the observed reduction in cell proliferation and confirming the key role of both receptors in the motility of TNBC cell lines. The obtained data supports the involvement of EGFR and IGF-IR in EMT-related gene regulation in cancer.

**Figure 7:**
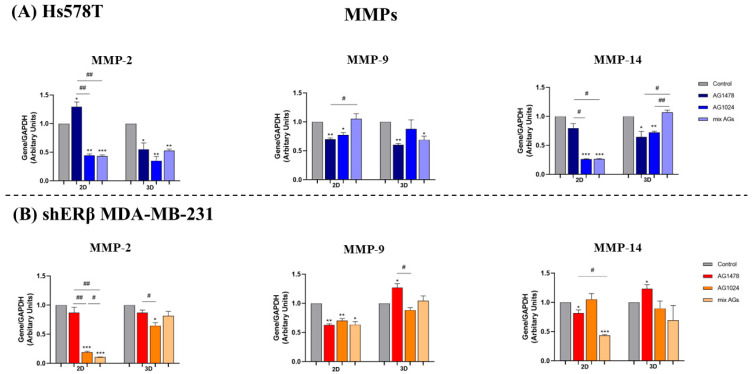
Effect of EGFR and IGF-IR inhibition on gene expression levels of MMPs in Hs578T and shERβ MDA-MB-231 in 2D and 3D cell cultures. (**A**) Quantification graphs of real-time PCR analysis of *MMP-2, -9* and *-14* mRNA levels in 2D and 3D cell cultures after treatment with the inhibitors AG1478, AG1024, and their combination, in Hs578T cells. (**B**) Quantification graphs of real-time PCR analysis of *MMP-2, -9* and *-14* mRNA levels in 2D and 3D cell cultures, after treatment with the inhibitors AG1478, AG1024, and their combination in shERβ MDA-MB-231 cells. Each bar represents mean ± SD values from triplicate samples. An asterisk (*) indicates statistically significant differences (*p* < 0.05), two asterisks (**) indicate statistically significant differences (*p* < 0.01) and three asterisks (***) indicate statistically significant differences (*p* < 0.001) compared to the control samples. A hash (#) indicates statistically significant differences (*p* < 0.05) and two hashes (##) indicate statistically significant differences (*p* < 0.01) between the different samples.

Another important regulator of breast cancer development is the expression level of MMPs, which are essential in facilitating tumor growth by influencing processes such as cell survival, proliferation, angiogenesis, and metastasis. Among them, the gelatinases MMP-2 and MMP-9 have been widely studied as potential biomarkers in breast cancer, with their overexpression consistently correlated with poor outcomes in several types of cancer. Furthermore, MMP-14 (MT1-MMP) is also related to poor prognosis in breast cancer patients and increased metastatic potential in multiple cancer models [41,42,43]. In the 2D cell cultures of Hs578T (Figure 7A), the combination of inhibitors resulted in a significant reduction in the expression levels of *MMP-2* and *MMP-14*, while the expression of *MMP-9* seemed to be influenced by the individual inhibition of each receptor. In 2D shERβ MDA-MB-231 cells (Figure 7B), a notable reduction in the expression of MMPs was observed, especially following the combinational treatment concerning *MMP-2* and *MMP-14*. Furthermore, cells derived from spheroids displayed a distinct pattern of MMP expression upon receptor inhibition. Interestingly, there were generally no significant statistical variances across most treatments, with the exception of increased *MMP-9* and *MMP-14* levels observed after the inhibition of EGFR. This increase may be linked to the enhanced migratory ability noted in these cells during the same treatment compared to their 2D equivalents. Conversely, in spheroid-derived cells, *MMP-2* was mainly affected by IGF-IR inhibition and combination of inhibitors, *MMP-9* was decreased by EGFR inhibition and the combination, while a decrease in *MMP-14* was observed following the individual inhibition of each receptor.

## 3. Discussion

In this study, the TNBC cell lines Hs578T and shERβ MDA-MB-231 (stable ERβ suppression) were incorporated due to their differing aggressiveness profiles and molecular characteristics, such as ERβ expression, allowing evaluation of treatment responses across distinct TNBC phenotypes in both 2D and 3D models. In previous studies by our research team, it was demonstrated that shERβ MDA-MB-231 cells exhibited a significant reduction in cell proliferation, in 2D monolayers, following EGFR inhibition [44]. Building on this, the use of 3D spheroid models further highlighted the critical roles of both EGFR and IGF-IR in sustaining tumor growth and proliferation under 3D conditions. These findings not only align with our earlier results but also clarify the involvement of IGF-IR in this process. Moreover, they are consistent with the well-established functions of both RTKs as key regulators of cell proliferation in breast cancer through the activation of their respective signaling pathways [20,45]. The dissemination assay underscored the advantages of using spheroid models for treatment evaluation, as they better simulate the early steps of tumor metastasis [30]. Notably, both receptors emerged as key promoters of this highly metastatic property in shERβ MDA-MB-231 cells, appearing to act synergistically. In contrast, only EGFR seemed to regulate cell dissemination in Hs578T cells, while the combination treatment showed no significant effect, but rather the addition of AG1024 stabilized it without reaching statistical significance, likely due to the activation of alternative mechanisms. A potential limitation of this study is that the presence of FBS during the dissemination assay, necessary to return spheroids to 2D conditions, combined with the rapid unbinding kinetics of AG1478 analogs such as Erlotinib [46], could have allowed partial reactivation of EGFR signaling. However, consistent with previous studies, the effects of AG1478 on functional properties are likely driven by specific EGFR inhibition, independent of serum supplementation [47,48]. Similarly, while inhibitors were applied according to established protocols for both EGFR and IGF-IR, direct confirmation of sustained receptor inhibition during prolonged incubations was not performed [49,50,51]. Nevertheless, the consistency of functional and transcriptional results across 2D and 3D models supports effective pathway targeting under the chosen conditions, although future studies could incorporate biochemical validation (e.g., Western blot) to further confirm receptor inhibition throughout extended assays.

The findings from the wound healing assays underscore the important role of RTKs in regulating the migratory capacity of breast cancer cells, particularly in TNBC [52]. In 2D monolayers, IGF-IR also emerges as a key driver of migration, especially in Hs578T cells, where its inhibition led to a more pronounced reduction in the migratory capacity, which was also translated into combinative inhibition. This suggests that, in Hs578T cells, IGF-IR may be the dominant regulator of migration, with no apparent synergistic effect when EGFR is also targeted. Additionally, both receptors appear to act cooperatively to promote migration in shERβ MDA-MB-231 cells, aligning with previous studies linking EGFR to cell motility in this cell line [44]. Conclusively, these patterns emphasize the functional relevance of both receptors in driving TNBC cell motility.

However, when these effects were evaluated in 3D spheroid-derived cells, the treatment responses differed from their 2D counterparts. In both Hs578T and shERβ MDA-MB-231 spheroid-derived cells, receptor inhibition did not significantly impact migration compared to untreated controls. This contrast highlights how 3D models, through their complex structure and microenvironment, can alter treatment response and cell behavior. Notably, shERβ MDA-MB-231 cells treated with the EGFR inhibitor exhibited a slightly increased wound closure compared to 2D cultures, suggesting a differential cellular response likely driven by the distinct conditions in 3D environments. These findings point to the importance of considering the dimensional context in treatment evaluation, as 3D models introduce cell–cell and cell–matrix interactions that can significantly influence therapeutic outcomes [53,54,55].

While our study primarily utilized morphological assessments to characterize 3D spheroid models, we acknowledge the importance of comprehensive structural and functional validation to enhance their physiological relevance. Future research should consider incorporating histological analyses, such as Hematoxylin and Eosin staining, to examine internal spheroid architecture, including the presence of necrotic cores and viable peripheral zones, which are characteristic of tumor histology [56]. Additionally, evaluating oxygen gradients within spheroids and validating hypoxic regions through immunostaining for hypoxia-inducible factor-1α (HIF-1α) and carbonic anhydrase IX (CAIX) would provide insights into the tumor microenvironment [57,58]. Assessing cell–cell adhesion integrity through immunohistochemical analysis of E-cadherin and β-catenin expression patterns is also recommended [59]. Furthermore, comparing spheroid invasion patterns during dissemination assays with invasion front characteristics observed in patient TNBC specimens could strengthen the translational applicability of these models. Addressing these aspects will not only validate the 3D spheroid models as legitimate tumor surrogates but also facilitate the development of more predictive preclinical platforms for evaluating therapeutic interventions in breast cancer.

**Figure 8:**
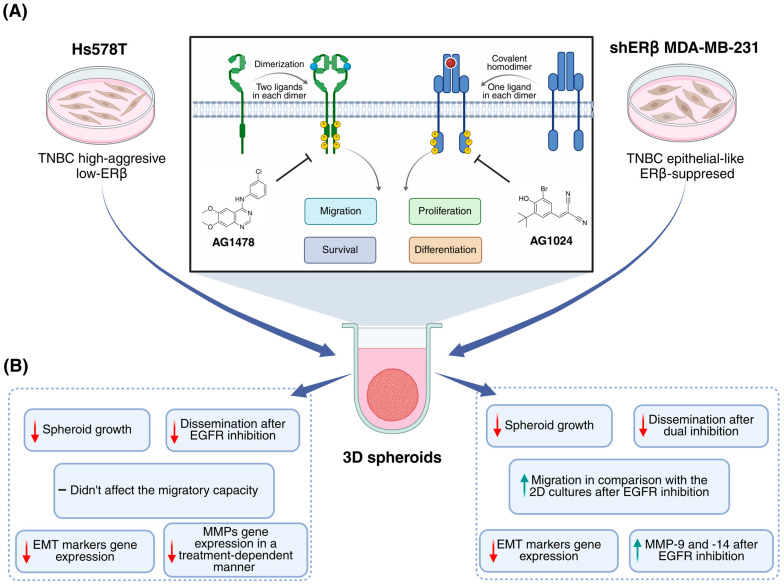
Targeting EGFR and IGF-IR in TNBC progression. (**A**) Among RTK family members, EGFR and IGF-IR are key regulators of breast cancer progression, promoting cellular responses such as proliferation, migration, survival, and differentiation through the activation of their respective signaling pathways. To better mimic the complex TME of solid tumors, 3D spheroids were incorporated using two distinct TNBC cell lines with differing levels of aggressiveness. The effect of inhibiting both receptors using their respective inhibitors, AG1478, AG1024 and their combination, was then evaluated in this culture model. (**B**) The most significant findings following receptor inhibition in both TNBC cell-derived spheroids are summarized. In the highly aggressive Hs578T cells, inhibition of both receptors led to a reduction in spheroid growth and EMT marker expression, while only EGFR inhibition appeared to significantly modulate dissemination. In the epithelial-like shERβ MDA-MB-231 cells, both receptors appear to be key regulators of spheroid growth, dissemination, and EMT marker expression. However, the migratory capacity was not affected by either treatment under 3D conditions and was even upregulated following EGFR inhibition in comparison with the 2D cell culture, accompanied with an elevated gene expression of *MMP-9* and *MMP-14*. Additionally, neither treatment affected migratory capacity under 3D conditions, although *MMPs* expression was downregulated in a treatment-dependent manner.

The bioinformatic analysis using the STRING database revealed that both receptors are strongly associated with the regulation of EMT progression and MMP activity at the protein level. Subsequent experimental evaluation of receptor inhibition in both 2D and 3D cell cultures further confirmed their critical role in modulating the gene expression of EMT markers. In particular, EGFR and IGF-IR emerged as key regulators of the mesenchymal markers *Slug* (*SNAI2*) and *vimentin* (*VIM*), reinforcing their role in promoting EMT in TNBC [44,55,60]. Additionally, in 2D conditions of both cell lines, the expression levels of *MMP-2* and *MMP-14* were significantly reduced following combined receptors inhibition, whereas *MMP-9* expression was primarily affected by the individual inhibition of each receptor. These findings align with the observed reduction in migratory capacity and EMT marker expression under treatment, as these MMPs are known as critical contributors to breast cancer progression [61]. In contrast, MMP expression in 3D conditions appeared to respond differently to receptor inhibition. Specifically, in Hs578T spheroids, a context-dependent reduction in MMP expression was observed, which did not correlate with the patterns seen in their corresponding 2D monolayers, highlighting further the distinct treatment responses in 3D environments. On the other hand, in shERβ MDA-MB-231 spheroids, only IGF-IR inhibition led to a reduction in *MMP-2* expression, while the expression of other MMPs remained largely unaffected across treatments. Notably, an opposite effect was observed for *MMP-9* and *MMP-14*, whose levels increased following EGFR inhibition, potentially explaining the enhanced migratory capacity under the same treatment, given the well-established roles of these MMPs in promoting cell migration [62,63]. Although the increase in *MMP-9* expression observed in 2D cultures after combined inhibition was not statistically significant, it may, together with the concomitant rise in *CDH1*, reflect crosstalk between EGFR/IGF-IR and other RTK family members. In this context, cells might attempt to maintain adhesion while simultaneously remodeling the ECM through through the action of proteolytic enzymes, such as MMP-9. Similarly, the increase in *MMP-14* expression detected under combined treatment in 3D spheroids may reflect altered, context-dependent responses specific to 3D systems. Figure 8 provides a summary of the dual receptor targeting concept in breast cancer (Figure 8A) and highlights the main results obtained from applying this approach in TNBC spheroid models of different aggressiveness (Figure 8B).

## 4. Materials and Methods

### 4.1. Cell Cultures and Reagents

The Hs578T (TNBC, high aggressive) and MDA-MB-231 (TNBC, ERβ+) breast cancer cell lines were purchased from the American Type Culture Collection (HTB-126, HTB-26, ATCC, Manassas, VA, USA). The development of shERβ MDA-MB-231 (ERβ-suppressed) was previously described by our laboratory team [54]. Both cell lines were cultured under stable conditions of 5% CO_2_ and 95% humidified air at a temperature of 37 °C. The cell culture medium was Dulbecco’s Modified Eagle’s Medium (DMEM, LM-D1110/500, Biosera, Cholet, France) and was supplemented with 10% fetal bovine serum (FBS, FB-1000/500, Biosera, France), as well as with antimicrobial agents (100 IU/mL penicillin, 100 μg/mL streptomycin, 10 μg/mL gentamycin sulfate and 2.5 μg/mL amphotericin B), and 2 mM L-glutamine (XC-T1715/100, Biosera, France). The cells were harvested at 80–85% cell confluency using trypsin-EDTA 1X in PBS (LM-T1706/500, Biosera, France). Dimethyl sulfoxide (DMSO) was used to prepare the stock solutions of the inhibitors Tyrphostin AG1478 (658552, Sigma Aldrich, Merck, Darmstadt, Germany) and Tyrphostin AG1024 (121767, Sigma Chemical Co., St Louis, MO, USA). All experiments were conducted under serum-free (0% FBS) conditions, to avoid net effects, in triplicate. The working concentrations of AG1478 and AG1024 were 2 μM and 1 μΜ, diluted in DMEM 0% FBS, respectively, and were chosen based on previous data from our group [44,51].

### 4.2. Development of 3D Spheroid Cultures

Breast cancer 3D spheroids were formed by culturing cells in 96-well round bottom plates with ultra-low adhesive properties (911606, SPL Life Sciences, Pocheon, South Korea). More precisely, Hs578T and shERβ MDA-MB-231 and cells were seeded at a density of 10,000–50,000 cells per well, based on the specific requirements of each experiment, and incubated for 72 h in complete medium DMEM with 10% FBS, without medium change, allowing for spheroid development. Spheroids were monitored through a contrast microscope (OLYMPUS CKX41, Olympus, Brooklyn Park, MN, USA) connected to a digital camera (QImaging MicroPublisher 3.3RTV, Virginia, QLD, Australia). Finally, the spheroids were either collected for total RNA extraction or transferred to standard flat-bottom well plates, for the evaluation of their functional properties.

### 4.3. Spheroid Growth and Proliferation

Following the formation of spheroids in complete medium (enhanced with 10% FBS), spheroids were starved with serum-free medium (0% FBS) for 16–20 h. Then, the inhibitors AG1478 (2 μΜ), AG1024 (1 μΜ) and their combination (AG1478 + AG1024) were added in serum-free medium for 24 h incubation. The spheroids were monitored only for 24 h to minimize confounding effects from secondary processes due to starvation or significant cell death. Subsequent analyses including morphological characterization and growth rate of the spheroids under treatment in comparison with the control spheroids (in serum-free medium), were made through a contrast microscope (OLYMPUS CKX41, USA) coupled with a digital camera (QImaging MicroPublisher 3.3RTV, Australia). For the proliferation assay, spheroids were washed three times with PBS, followed by the addition of trypsin-EDTA 1X in PBS for 10 min. Finally, they were transferred and diluted with complete medium DMEM (10% FBS) and were manually counted through a Neubauer hemocytometer for determination of their proliferation activity under treatment.

### 4.4. Spheroid Dissemination Assay

After the development of spheroids, a 16–20 h starvation time (DMEM, 0% FBS) took place with an additional 24 h incubation with AG1478 (2 μΜ), AG1024 (1 μΜ) and their combination (AG1478 + AG1024) in serum-free medium. Next, in order to evaluate the dissemination of spheroid-derived cancer cells, the already-treated spheroids were transferred using a pipette fitted with cut tips to prevent mechanical damage, to a regular, flat-bottom 48-well plate (1 spheroid/well) containing DMEM 5% FBS, to evaluate the impact of the inhibitors in cancer cell dissemination. Representative photos were captured using a phase-contrast microscope (OLYMPUS CKX41, USA) equipped with a digital camera (QImaging MicroPublisher 3.3RTV, Australia) at the time points 0, 24, 48, 72 and 96 h for each treatment. The obtained photos were further analyzed using ImageJ (1.5 ob Launcher Symmetry Software, LOCI, University of Wisconsin, USA) to quantify the area of dissemination [64]. The latter area was defined as the area covered by cells as they migrated away from the spheroid mass and was measured as inches^2^. All data were subsequently normalized to the corresponding values of 0 h for each experimental condition. As a result of this normalization, the units are effectively canceled, and the control is assigned a relative value of 1.

### 4.5. Wound Healing Assays in 2D and 3D Cell Cultures

In 2D cell cultures, Hs578T and shERβ-MDA-MB-231 cells were seeded into 48-well plates at a density of 1.5 × 10^4^ and 1.7 × 10^4^ cells per well, respectively, and incubated in complete medium (10% FBS) for 24 h. Then the medium was changed to serum-free (0% FBS), and the cells were starved for 16–20 h. The following day, the cell layer was wounded with a sterile 10 μL pipette tip in a straight line. Each well was washed twice with PBS to remove the detached cells. Then serum-free medium containing the cytostatic agent cytarabine (Sigma-Aldrich, Saint Louis, MO, USA, 10 μΜ) was added to eradicate the possibility of contribution of cell proliferation. After 30 min of incubation, the inhibitors AG1478 (2 μΜ), AG1024 (1 μΜ) and their mix (AG1478 + AG1024) were added to the respective well in serum-free (0% FBS) medium. The wound closure was observed at 0 and 24 h using a digital imaging camera (QImaging MicroPublisher 3.3RTV, Australia) connected to a phase-contrast microscope (OLYMPUS CKX41, USA). The effect of the inhibitors on cancer cell migration was measured by the quantification of wound surface area by image analysis (ImageJ 1.5 ob Launcher Symmetry Software, LOCI; University of Wisconsin) [64]. After spheroid development and treatment (16–20 h starvation, 24 h incubation with inhibitors in 0% FBS medium), 5 spheroids of each treatment condition were transferred using a pipette fitted with cut tips into a well of a 48-well plate containing DMEM (5% FBS). The cells were allowed to spread across the entire well over a period of 6 days, with the culture medium being refreshed every two days. Finally, when the cells reached the desired density, they were subjected to an additional starvation period and a fresh dose of inhibitors, ensuring drug presence during the subsequent wound-healing phase, as described above.

### 4.6. STRING Database

The STRING database is a bioinformatic platform that supports the creation and analysis of protein interaction networks for any genome of interest. The resulting network incorporates protein–protein interaction data, encompassing both direct (physical) interactions and indirect (functional) associations. The different types of interactions are derived from multiple sources, including text mining of scientific publications, computational predictions based on co-expression and conserved genomic context, experimentally verified interactions, and established complexes or pathways. The ‘Multiple proteins’ tool was used to construct the interaction network of the proteins of interest. This multifaceted approach ensures a robust representation of protein interaction networks, enabling the investigation of overlapping cellular activities and their potential roles in complex biological processes [65].

### 4.7. RNA Isolation, Complementary DNA (cDNA) Synthesis and Real-Time qPCR Analysis

For total RNA isolation in 2D cell cultures, Hs578T and shERβ MDA-MB-231 cells were cultured in complete medium (10% FBS), in 60 mm dishes at a density of 65 × 10^4^ and 60 × 10^4^ cells, respectively, for 24 h. Then the medium was switched to serum-free (0% FBS), and the cells were serum-starved for 16–20 h. The inhibitors AG1478, AG1024 and their mix (AG1478 + AG1024) were added in serum-free medium in concentrations of 2 and 1 μM, respectively, and the cells were incubated for 24 h. Finally, the next day, the cells were harvested using trypsin-EDTA 1X in PBS, followed by two washes with cold PBS 1X, each performed by centrifugation at 2400 rpm for 3 min. For total RNA isolation from 3D cell cultures, Hs578T- and shERβ MDA-MB-231-derived spheroids were cultured for 72 h in ultra-low-adhesion 96-well plates. Next, spheroids were starved (0% FBS) for 16–20 h and the inhibitors were added for 24 h incubation as described above. Afterwards, spheroids were collected using a 1000 μL pipette tip with a cut edge. On the following day, RNA was extracted from both 2D and 3D cell cultures, using the NucleoSpin^®^ RNA II Kit (Macherey-Nagel, Duren, Germany). To determine the RNA concentration and purity, absorbance was measured at 260 nm, and purity was evaluated using the 260/280 nm and 260/230 nm absorbance ratios. Complementary DNA (cDNA) synthesis was performed using the PrimeScript™ 1st strand cDNA synthesis kit perfect real time (Takara Bio Inc., Goteborg, Sweden). Real-time PCR (qPCR) was carried out using the KAPA Taq ReadyMix DNA Polymerase (KAPA BIOSYSTEMS, Wilmington, MA, USA), following the protocol provided by the manufacturer, in total volume of 20 μL per reaction. Amplification was executed on the Rotor Gene Q platform (Qiagen, Germantown, MD, USA). Each reaction was run in triplicate, and a standard curve was generated for every primer pair to confirm assay reliability. A melting curve analysis was also conducted to confirm specific amplification of the SYBR Green-labeled product. To analyze gene expression levels, the fluorescence signal was monitored during the exponential amplification phase, and the threshold cycle (Ct) value was identified by setting a fluorescence threshold above background levels. Relative gene expression was quantified using the ΔΔCt method, where Ct values of target genes were normalized against the housekeeping gene GAPDH. Fold changes in gene expression (reported in arbitrary units) were determined as 2^−ΔΔCt^. Information about the genes of interest and the corresponding primer sequences are presented in Table 1.

### 4.8. Statistical Analysis

All reported data are presented as the mean ± standard deviation (SD) from experiments performed in triplicate. Statistical significance was assessed using analysis of variance (ANOVA), followed by Tukey’s test to identify significant differences among the four experimental groups (control, AG1478, AG1024, and mixed AGs). Differences were considered statistically significant at the level of *p* ≤ 0.05, *p* ≤ 0.01 and *p* ≤ 0.001 indicated by (*, **, ***) for the treatment and control group comparison and by a hash sign (#, ##, ###) for the comparison between two different treatments. Statistical analysis and graphs were made using GraphPad Prism 8.0.1 (GraphPad Software, San Diego, CA, USA).

## 5. Conclusions

This study highlights the novel insights gained from using both 2D and 3D cell culture systems to investigate EGFR and IGF-IR function in TNBC. While 2D monolayers provided clear evidence of the receptors’ roles in regulating migration and EMT-related gene expression, the inclusion of 3D spheroid models revealed additional layers of complexity in treatment response, particularly in migratory behavior and matrix remodeling enzyme gene expression. These findings underscore the added value of 3D systems in capturing tumor-like architecture and TME, offering a more physiologically relevant context for future preclinical drug evaluation, which is in agreement with the prior literature demonstrating differences between 2D and 3D cultures and their impact on drug resistance [66]. Additionally, previous studies have already shown alterations in EGFR TKIs effectiveness between 2D and 3D cultures [67,68]. The present study extends this knowledge by demonstrating the particular importance of IGF-IR inhibition in 3D models and revealing that combining EGFR and IGF-IR targeted therapies may provide enhanced efficacy in TNBC. As a next step, our research will aim to further elucidate these phenomena through additional assays, focusing on signaling pathways, and matrix effectors, such as MMP activity, urokinase-type plasminogen activator (uPA), and cell surface proteoglycans, all of which are known to be implicated in metastatic potential. Building on this direction, although spheroids appear as valuable tools for drug screening in cancer research, the use of established cell lines limits their applicability in personalized medicine [69]. While TNBC cell lines are widely used and well-characterized models, they remain in vitro systems and do not fully represent the heterogeneity of clinical TNBC subtypes. Incorporating molecular profiling, such as the Lehmann classification system, could further enhance the translational relevance of these findings. The development of patient-derived spheroid cultures could represent the next step toward advanced personalized medicine and drug testing in breast cancer [70]. Ultimately, the incorporation of 3D spheroid models into preclinical evaluation systems may help bridge the gap between in vitro and in vivo studies, thereby enhancing the predictive value of drug testing in cancer research.

## Figures and Tables

**Table 1:** Primer sequences used for real-time polymerase chain reaction analysis.

Target Gene	Primers Sequences (5′–3′)		Annealing Τ (°C)
*SNAI2*	F	*AGACCCTGGTTGCTTCAAGGA*	60
	R	*CTCAGATTTGACCTGTCTGCAAA*	
*VIM*	F	*GGCTCGTCACCTTCGTGAAT*	60
	R	*GAGAAATCCTGCTCTCCTCGC*	
*CDH1*	F	*TACGCCTGGGACTCCACCTA*	60
	R	*CCAGAAACGGAGGCCTGAT*	
*MMP-2*	F	*CGTCTGTCCCAGGATGACATC*	62
	R	*ATGTCAGGAGAGGCCCCATA*	
*MMP-9*	F	*TTCCAGTACCGAGAGAAAGCCTAT*	62
	R	*GGTCACGTAGCCCACTTGGT*	
*MMP-14*	F	*CATGGGCAGCGATGAAGTCT*	60
	R	*CCAGTATTTGTTCCCCTTGTAGAAGTA*	
*GAPDH*	F	*AGGCTGTTGTCATACTTCTCAT*	60
	R	*GGAGTCCACTGGCGTCTT*	

## Data Availability

The raw data supporting the conclusions of this article will be made available by the corresponding authors on request.

## Abbreviations

The following abbreviations are used in this manuscript:

2D/3D	Two-/three-dimensional
BRCA1/2	Breast cancer gene 1/2
ECM	Extracellular matrix
EGFR	Epidermal growth factor receptor
EMT	Epithelial-to-mesenchymal transition
ERα/β	Estrogen receptor alpha/beta
HER2	Human epidermal receptor 2
IGF-IR	Insulin-like growth factor Ι receptor
MMPs	Matrix metalloproteinases
PR	Progesterone receptor
RTKs	Receptor tyrosine kinases
TME	Tumor microenvironment
TNBC	Triple-negative breast cancer
